# 

**Published:** 1889-09

**Authors:** 


### CONENTS—SEPTEMBER, 1889.—VOL. 5, NO. 3.

ORIGINAL COMMUNICATIONS— Page.
Ice, Natural or Artificial? W. M. Yandell, M. D. . . . . . 75
Diphtheria; with report of a case followed by paralysis. P. C. Coleman, M. D. . . . . . . . . . . . . . . . . . . 81
Summer Diarrhœa in Children. H. H. Thorpe, M. D. . . 83
An Anomalous Case of Midwifery. D. B. McGee, M. D. . 86
CORRESPONDENCE—
Inhalation in Whooping Cough. E. J. Beall, M. D. . . . 87
The Banquet Question and the P. M. B. A. A. D. Paulus, M. D . . . . . . . . . . . . . . . . . . . . . . 89
In Favor of a Banquet. B. W. Bristow, M. D . . . . . . 91
Blood-Letting in Tarantula Bite. T. J. Heard, M. D. . . 90
SOCIETY NOTES—
First Quarterly Meeting West Texas Medical Association; Minutes . . . . . . . . . . . . . . . . . . . . . 92
In Memoriam. Dr. J. T. Webb . . . . . . . . . . . . 93
Note: To the Doctors in the West Texas District. . . . . 94
Bosque County Medical Society Organized . . . . . . . . 95
New Tri-State Medical Society . . . . . . . . . . . . . 95
Southern Surgical and Gynecological Association . . . . 95
CULLINGS FROM CONTEMPORARIES—
Pharmacy in Mexico. R. P. Daniel, Chihuahua . . . . . 96
EDITORIAL—
The Scientifice Aspect of Brown-Sequrd's Discovery . . . 99
Important to County Medical Societies . . . . . . . . . . 101
Diphtheria . . . . . . . . . . . . . . . . . . . . . 103
A New and Valuable Surgical Instrument, of Texas Invention . . . . . . . . . . . . . . . . . . . . . . . . 107
Infantile Convulsions . . . . . . . . . . . . . . . . . . 108
MEDICAL NEWS AND MISCELLANY—
Numerous Paragraphs, wise and otherwise, and interesting more or less . . . . . . . . . . . . . . . . . . . . . . 109
PUBLISHER'S NOTES—
Which All Should Read . . . . . . . . . . . . . . . . . 116

